# Progress in Surgery

**Published:** 1897-05-08

**Authors:** 


### Progress in Surgery.

### HEAD INJURIES.

**Trade-like Movements following Head Injuries.**—In a paper read before the Edinburgh Medico-Chirurgical Society, Miles[1] records three cases in which patients, after sustaining severe head injuries, exhibited peculiar rhythmical and co-ordinated movements which, when carefully observed and analysed, turned out to be simply reproductions of the actions habitually gone through by them in the pursuit of their several trades. (1) A man fell from a roof 60 ft. high while following his occupation as a plumber. In addition to mistaking those around him for his fellow-workmen, the patient seemed to imagine himself at work, talked incoherently of it, and went through certain movements which one of his comrades voluntarily described as those of beating out lead. The right hand made short, sharp, hammering movements, while the left was constantly moving about as if smoothing down the lead. He became sensible later, but gradually sank, and died 75 hours after the injury. (2) A stone carver fell from a height of between 30 and 40 feet, striking the right side of his head against a projecting ledge as he fell. The spine was fractured about the level of the ninth dorsal vertebra. He was so violent that one-eightieth grain of hyoscine was injected to quieten him. When the effects of the drug passed off, it was observed that the constant movements of his arms were exactly those of a sculptor at work. This condition lasted for some hours, and then gave place to irregular restless movements and jerkings. He died ten days after the accident. (3) A man while painting a signboard fell to the ground and sustained severe head injuries. When still in a semi-sensible condition he continued to paint imaginary signs on a screen beside his bed, steadying the right hand, with which he made fine up and down movements on the opposite forearm. This patient recovered.

Remarking on these cases Miles states that the only records of similar movements he could find were those of Callender, published in 1867, who reported two cases On comparing the clinical symptoms with the morbid anatomy of the two fatal cases a striking parallelism becomes apparent. Both patients, at first, were in a state of violent excitement, constantly talking and shouting incoherently. Both were unable to recognise their friends and fellow-workmen, and persisted in addressing strangers by the names of their intimates. Each performed the movements appropriate to his trade. Although both subsequently died, in neither case was death due to compression or septic infection of the brain. The post-mortem appearances in the two cases were also strikingly similar. In each (1) the interior of the right occipital lobe was the seat of a vascular lesion; (2) several small petechial hæmorrhages had taken place into the grey matter of the angular gyris; (3) on the surface of the motor areas there were thin sub-arachnoid clots; (4) the substance of the frontal lobes (in the first case the left, and in the second the right) was the seat of petechial extravasations. In addition, in the first case, small effusions were found in the substance of the left tempero-sphenoidal lobe. The writer believes that the clinical phenomena are to be explained by a general disturbance of the function of cerebration, and that the grosser local lesions simply determined the direction in which this disturbance should be manifested. The anæmia of the brain following the injury left the nerve cells in an irritable and easily excited condition, and the cells, which most constantly and automatically discharge nerve energy, were the first to respond even to mild stimuli.

**Pistol-shot Injuries.**—Bullets in the cranial cavity have been localised by means of the Röntgen rays in two cases reported by Eulenburg.[2] (1) In one case a bullet entered $3\frac{1}{2}$ c.m. above and 2 c.m. in front of the attachment of the ear. From the clinical symptoms it was supposed that the bullet had penetrated to the right of the sella turcica, injuring the right optic tract and right crus. The X rays showed that the ball was in the middle fossa of the skull, to the right of the middle line. (2) In the other case the wound was self-inflicted ten years ago. Localising symptoms were few and indefinite, but the patient required to be kept for some years in an asylum. Although able for some work patient was suffering from headache, and was wasted. The bullet was located by radiography in the middle fossa of the skull, behind the right orbital fissure.

Another case is recorded by Brissaud and Londe,[3] where a bullet was localised in the posterior part of the skull at the level of the second temporal convolution, above the tentorium. This corresponded with the situation diagnosed by means of the clinical symptoms, and cleared up the nature of the hemiplegia which was present, and was due to a capsular and not a cortical lesion. An operation in these circumstances would have been useless.

In a case of suicidal pistol-shot wound of the skull, reported by Thelwall Thomas,[4] the bullet passed from side to side of the head without apparently entering the cranial cavity. On the right side the ball had entered at the outer side of the orbit, divided the optic nerve on that side, crossed the nasal cavity, and entered the left orbit. A counter opening was made at the outer angle of the left eye, but the bullet could not be found at the time. It was, however, found at the inner and lower part of the left orbit, and extracted a fortnight later. The whole track was drained by means of cyanide gauze. The patient recovered completely but for the blindness resulting from the division of the right optic nerve.

**Foreign Body in the Brain.**—A most interesting case, in which a piece of wood was extracted from the brain after having been there for thirty-two years without producing cerebral symptoms, is reported by Evans.[5] The patient, a soldier, during an engagement in 1862, was struck with what at the time was supposed to be a bullet on the left side of the face a little below the eye. No trouble followed, and he was ready for duty again in a week. For many years after leaving the army he laboured hard at agricultural work. A year before Evans saw him he was struck over the seat of the old cicatrix by a heavy batten; an abscess formed, and a discharging sinus was left. After various methods of treatment had failed to cure him Evans was consulted, and, opening up the sinus, found that it led through the posterior ethmoidal cells and the floor of the skull, exposing thickened dura water, through a small opening in which bloody pus escaped. A probe passed in came in contact with a foreign body, which, on extraction, proved to be a piece of pinewood measuring one and a quarter inches long, one-third of an inch wide, and one-third of an inch thick. The hæmorrhage was profuse, and necessitated plugging with gauze. Thirteen days after removal of the piece of wood the patient was seized with paralysis of the left side, with some thickness of speech. These symptoms passed off, and the patient ultimately resumed work.

**Traumatic Cerebral Abscess.**—Oppenheim[6] contributes an article on the differential diagnosis of cerebral abscess. Referring to those of traumatic origin, he points out that they have to be distinguished from (1) traumatic meningitis, (2) traumatic apoplexy, especially when this ensues some time after the accident, (3) traumatic encephalitis, (4) tumours of traumatic origin, (5) traumatic epilepsy, and (6) traumatic neurosis. Among the practical points to which he calls attention is the fact that in a certain proportion of cases distinct clinical evidence of cerebral hæmorrhage following an injury may not be present for some time after the accident. In one case of his own the symptoms did not appear for twenty days after the infliction of the injury. A very difficult diagnosis is that between late traumatic abscess and meningeal hæmorrhage followed by scar or cyst formation. The only certain signs are those peculiar to the onset of cerebral suppuration. A distinction must be made between suppurative and non-suppurative traumatic encephalitis. Traumatic hystero-neurasthenia is characterised by the absence of signs of increased intra-cranial tension, and the fact that the irritative or paralytic symptoms are usually on the same side as the injury. Friedman refers to a condition which sometimes follows head injuries—headache, vertigo, vaso-motor disturbances, loss of consciousness, with, sometimes, fever and irritability. This group of symptoms is due to minute pathological changes in the capillaries and arterioles of the brain. The writer has met with a case in which fatal tuberculous meningitis simulated traumatic cerebral abscess.

**Traumatic Meningocole.**—Rahm,[7] as a result of a study of the various methods of treating this condition, comes to the following conclusions: (1) In slight cases, compression, combined, if necessary, with tapping, may transform the hydrencephalocole into a mere hole in the skull. (2) In grave cases, the cavity of the meningocole should be extirpated, and the porencephalic cyst should be drained if necessary. Kronlein has operated on two cases by the latter method with satisfactory results. The greatest difficulty is found when the meningococle communicates with the ventricular cavities of the brain.

[1] Scot. Med. and Surg. Jour., March, 1897. [2] Deut. Med. Woch., Aug., 1897. [3] Sem. Méd., June, 1896. [4] Brit. Med. Journ., Oct. 1895. [5] Internat. Jour. Surg., 1896. [6] Berlin Klin. Wochen., No. 45, 1896. [7] Beiträge z. Klin. Chir., xvi., 1.

